# Suboptimal Handling of Piccolo Samples or Reagent Discs for Consideration in Ebola Response

**DOI:** 10.3201/eid2506.181928

**Published:** 2019-06

**Authors:** Jessica R. Spengler, Stephen R. Welch, Sarah C. Genzer, JoAnn Coleman-McCray, Jessica R. Harmon, Stuart T. Nichol, Christina F. Spiropoulou

**Affiliations:** Centers for Disease Control and Prevention, Atlanta, Georgia, USA

**Keywords:** clinical chemistry, field studies, outbreak response, mobile treatment unit, point-of-care, Ebola, Ebola virus disease, hemorrhagic fever, viruses

## Abstract

Operating clinical analyzers within recommended parameters can be challenging during outbreak response. Using the Piccolo Xpress point-of-care blood chemistry analyzer on guinea pig blood, we found that values of many analytes are still readily comparable when samples and reagent discs are handled at various conditions outside of manufacturer recommendations.

Blood chemistry analyses are useful for guiding patient care. However, following manufacturer-recommended handling and storage conditions can be challenging in areas with underdeveloped infrastructure, as experienced in past and ongoing Ebola outbreak response (*1*). To investigate the utility of data from samples or reagent discs handled under suboptimal conditions, we evaluated 14 conditions outside of manufacturer recommendations by using Strain 13/N guinea pig blood and plasma samples. Animal procedures were approved by the Centers for Disease Control and Prevention Institutional Animal Care and Use Committee and conducted at an AAALAC-International–accredited facility.

Samples were run on the Abaxis Piccolo Xpress Chemistry Analyzer (https://www.abaxis.com; quality control with Abbot General Chemistry controls and verification sample, https://www.fishersci.com/shop/products/pic-lpd-pls-gen-chm-ct-2x6x1ml/07p0401a). This platform is a compact and portable Clinical Laboratory Improvement Amendments–approved automated point-of-care system for whole blood, serum, and plasma (*2*). This platform, together with the General Chemistry 13 reagent disc used here, is widely used in past and ongoing Ebola outbreak responses (*3*–*6*) and in laboratory research on viral pathogenesis, therapeutics, and vaccine efficacy (*7*–*9*). All samples were collected in the recommended lithium heparin (LiH) tubes, except as indicated.

We determined intrinsic variation of each analyte under recommended conditions by running 31 samples on 2 different machines simultaneously or on 1 machine sequentially (represented as mean percentage change ± SD). We then evaluated 2 blood collection anticoagulants, 8 sample storage conditions, and 4 reagent disc storage conditions. Overall, >200 samples from 93 guinea pigs (48 males, 45 females; 7 were <1 month of age, 24 were 1–3 months, 8 were 4–6 months, 16 were 7–12 months, 17 were 1–2 years, 11 were 2–3 years, and 10 were >3 years), from healthy animals or animals with noninfectious chronic disease (e.g., renal failure) were analyzed.

We first evaluated the effect of anticoagulants on sample analytes. Blood was collected and then partitioned from the syringe into paired samples stored in LiH (baseline), EDTA, or sodium citrate. Deviations from baseline were determined and expressed as mean percentage differences (Table; Appendix [expanded color version]). As expected, because of the chelating action of EDTA, assay reactions involving cationic substrates (Ca^2+^, alkaline phosphatase) generated no values or were greatly altered, although values of several analytes remained closely comparable to baseline. Values from samples collected in sodium citrate were uniformly lower than those collected in LiH.

**Table:** Mean percentage change of clinical chemistry values obtained on the Piccolo Xpress Chemistry Analyzer from blood samples or reagent discs processed under various conditions that deviated from the manufacturer’s recommendations*

Condition	**No. tested**	**Percentage change compared with baseline**											
		GLU	BUN	CRE	CA	ALB	TP	ALT	AST	ALP	TBIL	GGT	AMY
Sample collection													
WB in EDTA†	15	−3.0	0.2	−0.8	ERR	4.1	−3.0	1.4	10.9	−93.2	−0.6	21.0	−1.7
WB in Na citrate†	18	−30.0	−23.3	−20.8	−59.1	−31.0	−34.4	−36.5	−23.6	−38.1	−6.9	−31.3	−0.5
Sample handling													
WB 4°C O/N†	13	−19.2	1.2	−9.4	−1.0	−2.3	0.0	1.6	20.1	−2.5	−1.9	227.3	0.0
WB RT O/N†	16	−76.4	1.7	−4.7	2.0	−4.2	4.9	−0.1	37.7	−9.3	0.6	572.3	1.2
WB 32°C O/N†	16	−92.6	8.9	6.4	0.7	−4.1	6.3	−0.2	64.0	−10.5	−1.8	489.0	2.8
WB −20°C O/N†	3‡	ERR	ERR	ERR	ERR	ERR	ERR	ERR	ERR	ERR	ERR	ERR	ERR
PL†	35	−0.4	1.1	13.2	1.2	1.1	0.1	2.8	−1.5	2.1	4.7	−11.1	−0.1
PL RT O/N§	15	0.4	2.9	−11.3	0.3	−4.1	1.9	−0.4	1.8	−3.3	−4.8	1.5	−0.5
PL 32°C O/N§	18	0.2	6.1	−12.2	0.1	−6.2	2.2	−5.5	2.1	−0.6	3.9	0.2	−0.5
PL −20°C O/N§	14	−1.1	0.4	−3.9	−2.4	−0.6	−1.6	2.5	4.0	−5.9	−3.6	0.0	−0.5
PL −20°C O/N + γ-irradiation§	16	0.3	3.4	11.3	2.5	0.0	−4.9	−12.3	−13.2	−15.8	1.6	−15.8	−0.5
Disc handling													
WB + disc RT 7 d†	15	−3.4	1.1	−3.6	−1.4	0.9	0.6	3.0	2.2	−2.9	2.2	36.6	1.2
WB + disc 32°C 5 d†	12	−4.3	0.5	56.7	0.3	1.3	0.8	−4.1	1.8	7.6	−7.5	22.0	1.7
WB + disc 32°C 14 d†¶	7‡	−0.9	ERR	ERR	−1.8	−8.0	−0.5	5.3	−0.2	ERR	0.0	0.5	−0.5
WB + disc 32°C 5 wk†	2‡	ERR	ERR	ERR	ERR	ERR	ERR	ERR	ERR	ERR	ERR	ERR	ERR
Intrinsic variation													
±1 SD		3.0	2.8	18.3	1.5	3.1	1.8	8.9	4.0	13.2	16.6	20.3	1.8
±2 SD		5.9	5.7	36.5	3.1	6.2	3.7	17.7	8.1	26.5	33.3	40.6	3.7
Reference values#		143 ± 16 mg/dL	20.5 ± 3.3 mg/dL	0.32 ± 0.2 mg/dL	11.4 ± 0.5 mg/dL	2.9 ± 0.2 g/dL	5.4 ± 0.3 g/dL	27 ± 6 U/L	46 ± 15 U/L	43 ± 15 U/L	0.3 ± 0.04 mg/dL	10 ± 3 U/L	1,149 ± 131 U/L

*All analytes were quantified with the Piccolo General Chemistry 13 reagent discs (https://www.abaxis.com). Baseline values were obtained from aliquots of the same samples run according to manufacturer’s recommendations for comparison. Values in white cells varied by <1 SD; values in light gray cells, by 1–2 SD; and values in dark gray cells, by >2 SD from the determined % intrinsic variation derived from analysis of samples run either sequentially on the same machine or in parallel on different machines. γ indicates γ-irradiated at 5 × 10^6^ rads. ALB, albumin; AMY, amylase; ALP, alkaline phosphatase; ALT, alanine aminotransferase; AST, aspartate aminotransferase; BUN, blood urea nitrogen; CA, calcium; CRE, creatinine; ERR, analyte, sample, or disc error; GGT, γ-glutamyltransferase; GLU, glucose; Na, sodium; O/N, overnight; PL, plasma; RT, room temperature; TBIL, total bilirubin; TP, total protein; WB, whole blood. An expanded version of this table is available online (https://wwwnc.cdc.gov/EID/article/25/6/18-1928-T1.htm). †Baseline sample: WB. §‡Smaller sample size tested because tests did not function at indicated condition.  ‡§Baseline sample: PL. ¶Only subset of discs (7 of 10) generated values; remainder generated no values because of disc error. #Reference values for strain 13/N guinea pigs 50–900 d of age, expressed as mean ± SD (*10*).

To assess effects of sample storage temperatures on data output, we conducted similar analyses on whole blood stored overnight at −20°C, −4°C, or room temperature (≈20°C–22°C) and on plasma stored overnight at −20°C, room temperature, or 32°C. In general, plasma was more resistant to suboptimal temperatures; values for most analytes were comparable with paired control. Although data from whole blood samples varied more, many analytes (creatinine, alanine aminotransferase, alkaline phosphatase, total bilirubin) remained within ± 1 SD of baseline values. This finding is useful in situations where centrifuging blood samples might not be possible. For application to high-containment studies, we also evaluated the utility of data from plasma stored overnight at −20°C and γ-irradiated (5 × 10^6^ rads) before analysis. Under these conditions, 6 of 12 analytes varied by >1 SD from expected range, suggesting that samples should be processed before inactivation by γ-irradiation.

Finally, we assessed effects of storing reagent discs at room temperature or 32°C for varying lengths of time. Although discs must be refrigerated when stored >48 hours, all values obtained from discs stored at room temperature for a week were within ± 1 SD of controls, except glucose and γ-glutamyltransferase (± 1–2 SD). Data from discs stored at 32°C for 5 days were similarly comparable with baseline; we observed deviations in glucose, γ-glutamyltransferase, and creatinine. Discs stored at 32°C for 14 days were unreliable; many generated no values or a “disc error” message. However, values read from discs that did not result in an error message were all within expected limits. All discs stored at 32°C for 5 weeks generated error messages. These findings suggest that although extended storage at elevated temperatures substantially damages discs, discs stored at these conditions for up to 2 weeks might still yield clinically relevant data.

In summary, we found that under various suboptimal conditions, many analytes are still readily comparable (within ± 1 SD of intrinsic variation) to those from paired samples handled according to the manufacturer’s recommendations. In addition, the pattern of values that deviate from baseline is often consistent (e.g., glucose decreases) and may be considered in clinical evaluation. Although we used SDs to highlight deviation here, the clinical implication of these findings will vary based on the analyte and condition being monitored. We believe these findings, based on guinea pig samples, enabling us to efficiently sample a large population and process under controlled conditions, are translatable to other species, including humans, because the equivalent veterinary point-of-care platform (VetScan VS2) is also disc-based and uses comparable assay chemistry. However, the possibility for some species differences remains and should be considered for future investigations.

Appendix Results of clinical chemistry values obtained on the Piccolo Xpress Chemistry Analyzer from blood samples processed under various conditions.
