# Supplementary material for: Suboptimal Handling of Piccolo Samples or Reagent Discs for Consideration in Ebola Response
Source: Emerg Infect Dis. 2019 Jun;25(6):1238–40. doi: 10.3201/eid2506.181928 (PMC6537746; doi:10.3201/eid2506.181928)
Supplement: Appendix — Results of clinical chemistry values obtained on the Piccolo Xpress Chemistry Analyzer from blood samples processed under various conditions. [file 18-1928-Techapp-s1.pdf]

Article DOI: https://doi.org/10.3201/eid2506.180296

Suboptimal Handling of Piccolo Samples or Reagent Discs for Consideration in Ebola Response

Appendix

Appendix Table. Percentage change of clinical chemistry values obtained on the Piccolo Xpress Chemistry Analyzer from blood samples or reagent discs processed under various conditions deviating from manufacturer's recommendations*

Condition	No. tested	GLU	BUN	CRE	CA	ALB	TP	ALT	AST	ALP	TBIL	GGT	AMY
Sample collection													
WB in EDTA†	15	−3.0 (−13.8 to 7.0)	0.2 (−5.6 to 6.3)	−0.8 (−33.3 to 50.0)	ERR	4.1 (−3.7 to 12.5)	−3.0 (−10.9 to 1.9)	1.4 (−11.8 to 11.4)	10.9 (−28.8 to 51.9)	−93.2 (−94.3 to −92.4)	−0.6 (−25.0 to 33.3)	21.0 (−75.9 to 100.0)	−1.7 (−5.2 to 4.9)
WB in Na citrate†	18	−30.0 (−55.5 to −22.5)	−23.3 (−40.9 to −14.3)	−20.8 (−50.0 to 0.0)	−59.1 (−63.4 to −55.4)	−31.0 (−53.6 to −25.0)	−34.4 (−59.6 to −29.6)	−36.5 (−65.5 to −15.4)	−23.6 (−47.6 to 13.3)	−38.1 (−52.3 to −13.9)	−6.9 (−33.3 to 33.3)	−31.3 (−51.7 to −19.2)	−0.5 (−0.8 to −0.3)
Sample handling													
WB 4°C O/N†	13	−19.2 (−38.5 to −7.7)	1.2 (−5.0 to 5.9)	−9.4 (−33.3 to 50.0)	−1.0 (−4.3 to 4.9)	−2.3 (−13.3 to 3.4)	0.0 (−2.1 to 2.3)	1.6 (−26.5 to 25.8)	20.1 (9.1 to 48.3)	−2.5 (−12.7 to 3.2)	−1.9 (−25.0 to 0.0)	227.3 (50.0 to 750.0)	0.0 (−3.8 to 6.4)
WB RT O/N†	16	−76.4 (−93.2 to −39.1)	1.7 (−5.9 to 5.9)	−4.7 (−57.1 to 66.7)	2.0 (−5.0 to 6.2)	−4.2 (−10.0 to 4.5)	4.9 (0.0 to 13.0)	−0.1 (−14.3 to 28.6)	37.7 (14.8 to 97.5)	−9.3 (−65.0 to 9.1)	0.6 (−25.0 to 33.3)	572.3 (55.6 to 2328.6)	1.2 (−4.5 to 8.3)
WB 32°C O/N†	16	−92.6 (−93.5 to −91.8)	8.9 (0.0 to 15.8)	6.4 (−60.0 to 66.7)	0.7 (−5.4 to 4.5)	−4.1 (−18.4 to 3.2)	6.3 (2.0 to 15.7)	−0.2 (−26.9 to 25.7)	64.0 (−29.6 to 207.5)	−10.5 (−35.6 to 1.3)	−1.8 (−25.0 to 0.0)	489.0 (0.0 to 750.0)	2.8 (1.5 to 4.9)
WB −20°C O/N†	3§	ERR	ERR	ERR	ERR	ERR	ERR	ERR	ERR	ERR	ERR	ERR	ERR
PL†	35	−0.4 (−7.7 to 5.4)	1.1 (−7.1 to 21.7)	13.2 (−33.3 to 100.0)	1.2 (−3.1 to 4.5)	1.1 (−3.8 to 11.1)	0.1 (−3.9 to 5.8)	2.8 (−10.8 to 40.0)	−1.5 (−23.2 to 76.7)	2.1 (−59.3 to 31.3)	4.7 (−25.0 to 33.3)	−11.1 (−66.7 to 62.5)	−0.1 (−4.2 to 2.7)
PL RT O/N‡	15	0.4 (−1.5 to 4.7)	2.9 (−2.0 to 8.7)	−11.3 (−40.0 to 50.0)	0.3 (−11.2 to 5.0)	−4.1 (−13.0 to 4.0)	1.9 (0.0 to 5.8)	−0.4 (−11.5 to 7.1)	1.8 (−5.7 to 9.1)	−3.3 (−25.6 to 19.4)	−4.8 (−25.0 to 33.3)	1.5 (−1.7 to 5.1)	−0.5 (−0.8 to −0.3)
PL 32°C O/N‡	18	0.2 (−4.1 to 4.4)	6.1 (0.0 to 15.4)	−12.2 (−40.0 to 50.0)	0.1 (−19.6 to 3.6)	−6.2 (−13.8 to 3.7)	2.2 (−3.7 to 6.1)	−5.5 (−19.4 to 23.5)	2.1 (−6.1 to 15.0)	−0.6 (−22.2 to 33.3)	3.9 (0.0 to 33.3)	0.2 (−4.0 to 4.5)	−0.5 (−0.8 to −0.3)
Plasma −20°C O/N‡	14	−1.1 (−5.0 to 1.7)	0.4 (−5.0 to 5.3)	−3.9 (−40.0 to 33.3)	−2.4 (−7.7 to 0.0)	−0.6 (−13.0 to 8.3)	−1.6 (−5.8 to 2.2)	2.5 (−16.7 to 35.3)	4.0 (−5.7 to 16.1)	−5.9 (−31.4 to 25.6)	−3.6 (−25.0 to 0.0)	0.0 (−3.5 to 4.5)	−0.5 (−0.8 to −0.3)
PL −20°C O/N + γ-irradiation‡	16	0.3 (−3.0 to 10.6)	3.4 (−2.0 to 13.6)	11.3 (−50.0 to 100.0)	2.5 (−5.6 to 10.5)	0.0 (−13.0 to 11.1)	−4.9 (−90.6 to 13.7)	−12.3 (−42.3 to 19.0)	−13.2 (−20.8 to 0.0)	−15.8 (−38.1 to 23.1)	1.6 (−25.0 to 33.3)	−15.8 (−19.7 to −5.2)	−0.5 (−0.8 to −0.3)
Disc handling													
WB + disc RT 7 d†	15	−3.4 (−6.0 to −0.7)	1.1 (−4.5 to 11.1)	−3.6 (−60.0 to 66.7)	−1.4 (−2.6 to 0.0)	0.9 (−3.3 to 9.7)	0.6 (−2.0 to 3.9)	3.0 (−19.2 to 18.2)	2.2 (−13.2 to 21.9)	−2.9 (−19.7 to 8.3)	2.2 (0.0 to 3.3)	36.6 (−16.7 to 183.3)	1.2 (−1.2 to 6.8)
WB + disc 32°C 5 d†	12	−4.3 (−11.8 to 1.4)	0.5 (−5.0 to 6.3)	56.7 (−25.0 to 150.0)	0.3 (−0.9 to 1.8)	1.3 (−3.7 to 7.4)	0.8 (−2.4 to 3.9)	−4.1 (−19.5 to 30.6)	1.8 (−7.3 to 11.4)	7.6 (−4.4 to 44.8)	−7.5 (−25.0 to 0.0)	22.0 (−15.4 to 66.7)	1.7 (−1.0 to 5.1)
WB + disc 32°C 14 d¶	7§	−0.9 (−4.1 to 0.7)	ERR	ERR	−1.8 (−2.6 to −0.9)	−8.0 (−12.0 to −6.5)	−0.5 (−1.8 to 0.0)	5.3 (−5.6 to 17.6)	−0.2 (−2.2 to 3.6)	ERR	0.0 (0.0 to 0.0)	0.5 (−6.3 to 7.7)	−0.5 (−0.8 to −0.3)
WB + disc 32°C 5 wk†	2§	ERR	ERR	ERR	ERR	ERR	ERR	ERR	ERR	ERR	ERR	ERR	ERR
Intrinsic variation													
±1 SD		±3.0	±2.8	±18.3	±1.5	±3.1	±1.8	±8.9	±4.0	±13.2	±16.6	±20.3	±1.8
±2 SD		±5.9	±5.7	±36.5	±3.1	±6.2	±3.7	±17.7	±8.1	±26.5	±33.3	±40.6	±3.7
Reference values#		143 ± 16 mg/dL	20.5 ± 3.3 mg/dL	0.32 ± 0.2 mg/dL	11.4 ± 0.5 mg/dL	2.9 ± 0.2 g/dL	5.4 ± 0.3 g/dL	27 ± 6 U/L	46 ± 15 U/L	43 ± 15 U/L	0.3 ± 0.04 mg/dL	10 ± 3 U/L	1,149 ± 131 U/L

*All analytes were quantified with the Piccolo General Chemistry 13 reagent discs (https://www.abaxis.com). Baseline values were obtained from aliquots of the same samples run according to manufacturer's recommendations for comparison. Values represent mean and range of percentage change. Mean values in green cells varied by <1 SD; mean values in blue cells, by 1–2 SD; and mean values in red cells, by >2 SD from the determined % intrinsic variation derived from analysis of samples run either sequentially on the same machine or in parallel on different machines. γ indicates γ-irradiated at 5×10^6 rads.ALB, albumin; AMY, amylase; ALP, alkaline phosphatase; ALT, alanine aminotransferase; AST, aspartate aminotransferase; BUN, blood urea nitrogen; CA, calcium; CRE, creatinine; ERR, analyte, sample, or disc error; GGT, γ-glutamyltransferase; GLU, glucose; Na, sodium; O/N, overnight; PL, plasma; RT, room temperature; TBIL, total bilirubin; TP, total protein; WB, whole blood.
†Baseline sample: WB
‡Baseline sample: PL
§Smaller sample size tested because tests did not function at indicated condition.
¶Only subset of discs (7 of 10) generated values; remainder generated no values because of "disc error" (ERR).
#Reference values for strain 13/N guinea pigs aged 150–900 d, expressed as mean ± SD (*10*).
